# Case report: Forced walking for treating lower limb paralysis after corpus callosum injury

**DOI:** 10.1097/MD.0000000000048767

**Published:** 2026-05-22

**Authors:** Xiaodong Xu, Shiqiang Zhang, Chencong Gu, Wei Feng, Xiao Wang, Wenqian Bi

**Affiliations:** a Hebei Province Key Laboratory of Integrated Traditional and Western Medicine in Neurological Rehabilitation, Cangzhou Hospital of Integrated Traditional Chinese and Western of Hebei Province, Cangzhou, Hebei, China.

**Keywords:** case report, cerebral infarction, corpus callosum, diffusion tensor imaging, high walk assistive device

## Abstract

**Rationale:**

Damage to the corpus callosum induced by cerebral infarction is a relatively uncommon injury. Patients may exhibit diminished limb sensorimotor integration and reduced muscle strength, which makes rehabilitation more difficult. We aim to present a case of long-term paralysis in 1 lower limb resulting from severe damage to the corpus callosum fiber bundles following a cerebral infarction, and to demonstrate effective rehabilitation training methods.

**Patient concerns:**

The patient experienced a cerebral infarction 2 hours after pituitary tumor surgery. Two months after undergoing conventional rehabilitation training, the right lower limb was still paralyzed.

**Diagnoses:**

A diffusion tensor imaging examination revealed that the corpus callosum fiber bundle and corticospinal tract were severely damaged, which explains the slow recovery. The diffusion tensor imaging results showed that the patient had insufficient muscle strength in their right lower limb and weak lower limb sensorimotor integration.

**Interventions:**

We performed forced walking training with the assistance of a high walk assistive device. This method requires the use of both limbs in a symmetrical manner and forces the patient to concentrate, thereby increasing the sensorimotor integration of the affected limb.

**Outcomes:**

After 2 weeks of rehabilitation training, the patient achieved independent walking under supervision.

**Lessons:**

Using a high walk assistive device for forced walking training may improve the lower limb motor function of patients with corpus callosum injury. A manual muscle testing grade of 0 may mask residual motor capacity in some patients.

## 1. Introduction

Postoperative hemodynamic changes, thrombosis, and anesthesia-related factors may cause a cerebral infarction. Patients who have had a cerebral infarction may experience movement, speech, swallowing, and cognitive disorders.^[1]^ These disorders reduce patients’ ability to perform daily activities, negatively impacting their quality of life.^[2]^ They also prevent patients from resuming normal life and work activities. The corpus callosum is the largest white matter fiber bundle and connects the left and right cerebral hemispheres. Consisting of a large number of nerve fibers, it is divided into 4 sections: the mouth, knee, body, and compression section. The corpus callosum primarily facilitates nerve conduction between corresponding regions of the 2 hemispheres. It plays an important role in maintaining integrated brain function and coordinating limb movement.^[3]^ The corpus callosum receives a dual blood supply from the anterior and posterior circulations of the brain. For this reason, the incidence of corpus callosum damage in cerebral infarction is very low.^[4]^

A retrospective study found that the incidence of corpus callosum infarction was 2.3%. Even after 24 hours, the negative imaging tomography rate was 76.4%.^[5]^ Magnetic resonance imaging may have limited diffusion in the corpus callosum, which can lead to uncertainty in diagnosis. Studies have found that over half of lesions in the corpus callosum are caused by factors other than vascular issues, such as tumors and demyelinating changes. These patients may need to undergo further diagnostic tests.^[6]^

The clinical manifestations of corpus callosum damage include apraxia, alien hand syndrome, hypesthesia, speech disorders, and cognitive impairment, among others. Among them, lower limb dyskinesia can be manifested as lower limb weakness, sitting instability, and difficulty standing and walking. Patients with severely damaged corpus callosum often have more difficulty regaining independent community walking skills. For patients, being able to walk independently again is an important rehabilitation goal.^[7]^ We usually use conventional exercise rehabilitation training to address poststroke movement disorders. This includes roll-over training, trunk core training, sitting-up training, standing training, weight-transfer training, and balance training.^[8]^ However, after 2 months of routine rehabilitation, the patient did not regain balance or walking ability. Through a literature review, we found that current research on corpus callosum damage primarily focuses on clinical manifestations and imaging features. Effective rehabilitation training methods are lacking. Therefore, it is necessary to explore new methods for treating dyskinesia caused by corpus callosum damage.

## 2. Patient information

On February 16, 2025, a 54-year-old male patient underwent a neuroendoscopic transnasal transsphenoidal resection of a pituitary lesion under general anesthesia. Two hours after the operation, the patient was unable to move the right limb, had difficulty speaking fluently, and experienced decreased sensation of pain, as well as decreased memory and computational ability. Diffusion-weighted imaging revealed acute cerebral infarctions in the left frontal and parietal lobes. After 15 days of treatment in the Department of Neurology, the right upper limb was inflexible, and the lower limb could not move.

The patient has had hypertension for about 20 years. The history of pituitary adenoma was found to be about 7 years. The patient denied a chronic history of diabetes or coronary heart disease. The patient denied a family history of genetic disease.

## 3. Clinical findings

The patient was transferred to the Neurorehabilitation Department on March 3, 2025. Physical examination findings: clear consciousness, less fluent speech, and normal listening comprehension. The bilateral frontal stria is symmetrical without thinning, and the bilateral nasolabial sulcus is symmetrical. Brunnstrom classification (right upper limb, hand lower limb): IV–IV–I. Sitting balance: level 2/3. Standing balance: not established. Medical Research Council muscle scale: The muscle strength of the proximal right upper limb is 3+/5, the muscle strength of the distal right upper limb is 4−/5, and the muscle strength of the right lower limb is 0/5. Modified Ashworth Scale: grade 0/5 for both the right upper and lower limbs. The muscle strength and tension in the left limb are normal. The sensation of pain in the right limb decreased. No abnormalities were found during deep sensory examination. The right biceps brachii tendon reflex is negative (−). The right triceps brachii tendon reflex is negative (−). The right knee tendon reflex is active (++). The right Hoffman sign is positive (+). The right Babinski sign is positive (+). The Holden Walking Function Grade was 0/5. The modified Barthel Index score was 33. The Mini-Mental State Examination score was 21.

## 4. Timeline

**Figure 1.**
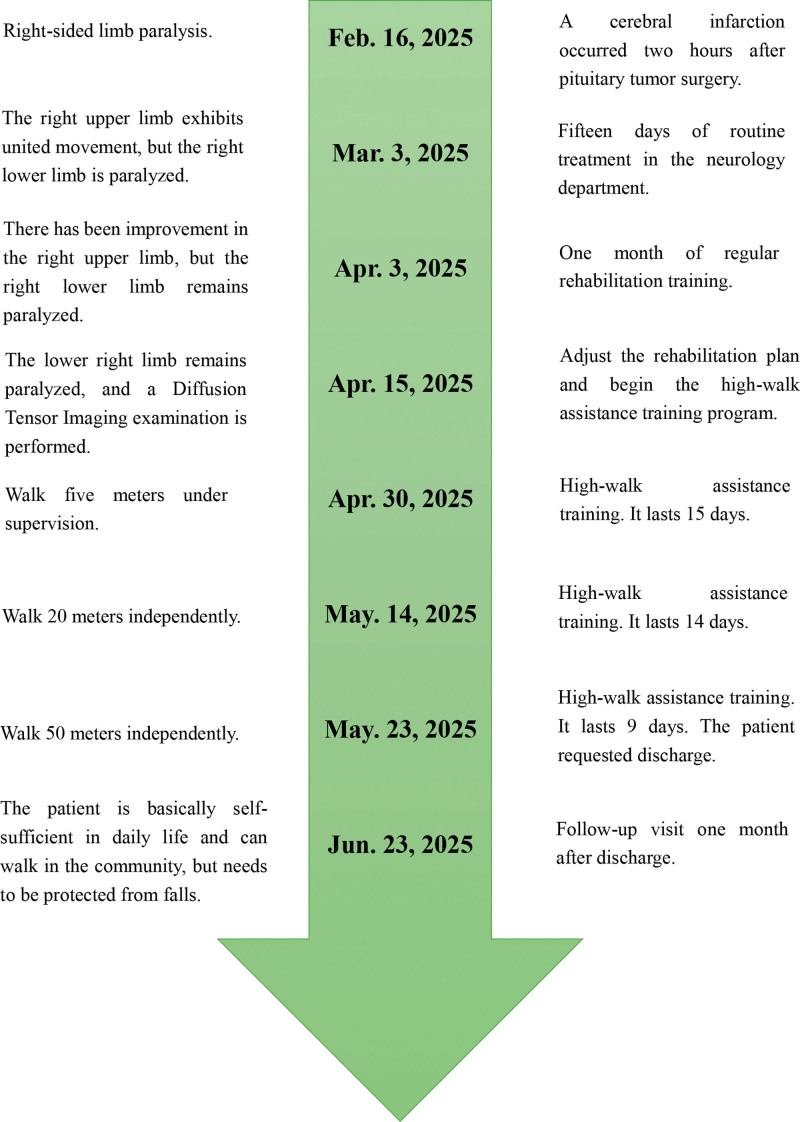
Patient treatment timeline.

On February 16, 2025, the patient suffered a cerebral infarction following a surgical procedure. On March 3, 2025, he was transferred to the Neurorehabilitation Department for treatment. Forty-three days later, there was still no improvement in his paralyzed lower limb. On April 15, 2025, the patient underwent a diffusion tensor imaging (DTI) examination and then began walking training with the assistance of a high walk assistive device. After 38 days of treatment, the patient could walk 50 meters independently, though they exhibited a foot-drop gait. The patient requested to be discharged to their home. One month after being discharged, the patient could walk in the community but still needed help to prevent falls (Fig. 1).

## 5. Diagnostic assessment

**Figure 2.**
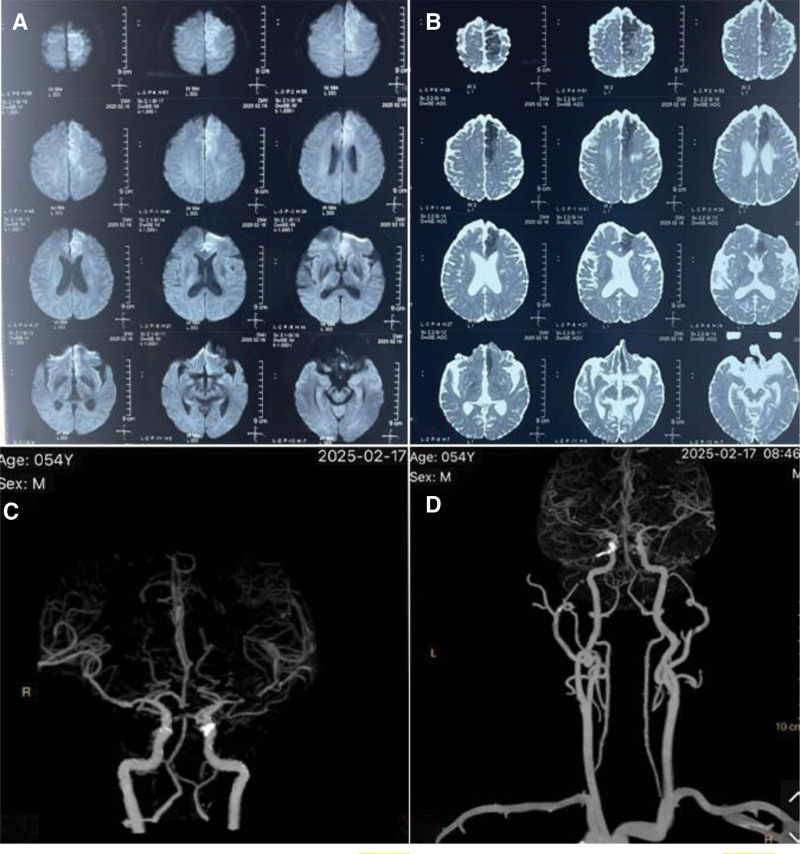
(A) Diffusion-weighted imaging. (B) Apparent diffusion coefficient image. (C) CTA of the head. (D) CTA of the head and neck. CTA = computed tomography angiography.

On February 16, 2025, brain functional imaging (field strength ≥ 3T) showed cerebral infarction near the midline of the left frontal lobe and left radiation coronal malacia (Fig. 2A and B). A computed tomography angiography scan of the head and neck performed on February 17, 2025, revealed the following: calcified plaques at the beginning of the left common carotid artery, at the end of the bilateral common carotid arteries, and at the beginning of the bilateral internal carotid arteries. There was mild stenosis of the local lumen. There were calcified plaques in the siphon of the bilateral internal carotid arteries, with mild stenosis of the local lumen. The M1 segment of the left middle cerebral artery was severely stenosed and tended to occlude. The local lumen of the A1 segment of the left anterior cerebral artery was severely stenotic and tended to occlude. The local lumen of the M1 segment of the right middle cerebral artery was enlarged (Fig. 2C and D).

**Figure 3.**
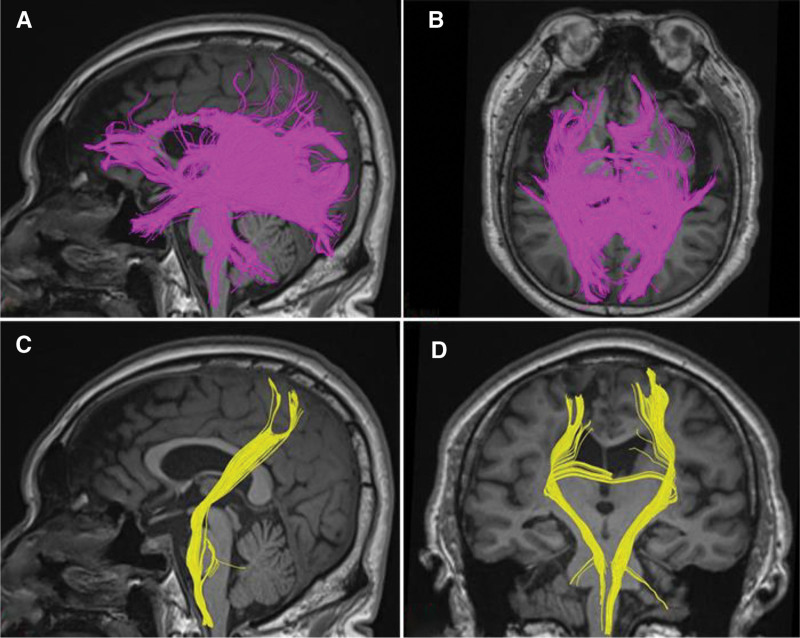
(A) Left side view of the corpus callosum. (B) Superior view of the corpus callosum. (C) Left side view of the corticospinal tract. (D) Frontal view of the corticospinal tract.

DTI inspection (April 15, 2025) showed that the nerve fibers in the mouth, knee, and the anterior half of the corpus callosum were clearly damaged. Most of these fibers did not reach the cortex, and the anterior commissural fiber bundles were sparse. The left corticospinal tract was partially interrupted at the pontine level, and brain network connections were reduced (Fig. 3).

Scanning equipment: 3.0 Tesla magnetic resonance imaging scanner (model: Siemens Prisma; Siemens Healthineers AG). Patients were fitted with standard head coils. During scanning, secure the head with foam pads to minimize motion artifacts. Provide earplugs and noise-canceling headphones. Scan sequence: single-shot spin-echo Echo Planar Imaging. DTI parameters: voxel size 2.0 × 2.0 × 2.0 mm^3^. *B*-value: 1000 s/mm^2^. Number of diffusion directions: 64. The analysis was performed using the DSI Studio software (http://dsi-studio.labsolver.org). The evaluation criteria were as follows: The analysis was conducted based on regions of interest within the target brain areas. The regions of interest were delineated using a standard spatial atlas, which enabled the reconstruction and visualization of specific fiber tracts passing through them, such as corticospinal and corpus callosum fibers.

Diagnosis: cerebral infarction recovery period.

## 6. Therapeutic intervention

After the patient was transferred to the Neurorehabilitation Department, the prescribed medications included amlodipine besylate tablets (5 mg, once a day), urapidil sustained-release tablets (30 mg, once a day), indobufen tablets (0.1 g, twice a day), and benzbromarone tablets (25 mg, once a day). The patient received physical and occupational therapy. This included learning how to turn over, sit up, stand, shift their center of gravity, and do aerobic exercises. They did this for 40 minutes, once a day. They also received the following treatments: acupuncture (for 30 minutes, once a day), massage (for 30 minutes, once a day), medium-frequency electrical stimulation (for 15 minutes, once a day), and repetitive transcranial magnetic stimulation treatments (for 15 minutes, once a day).

**Figure 4.**
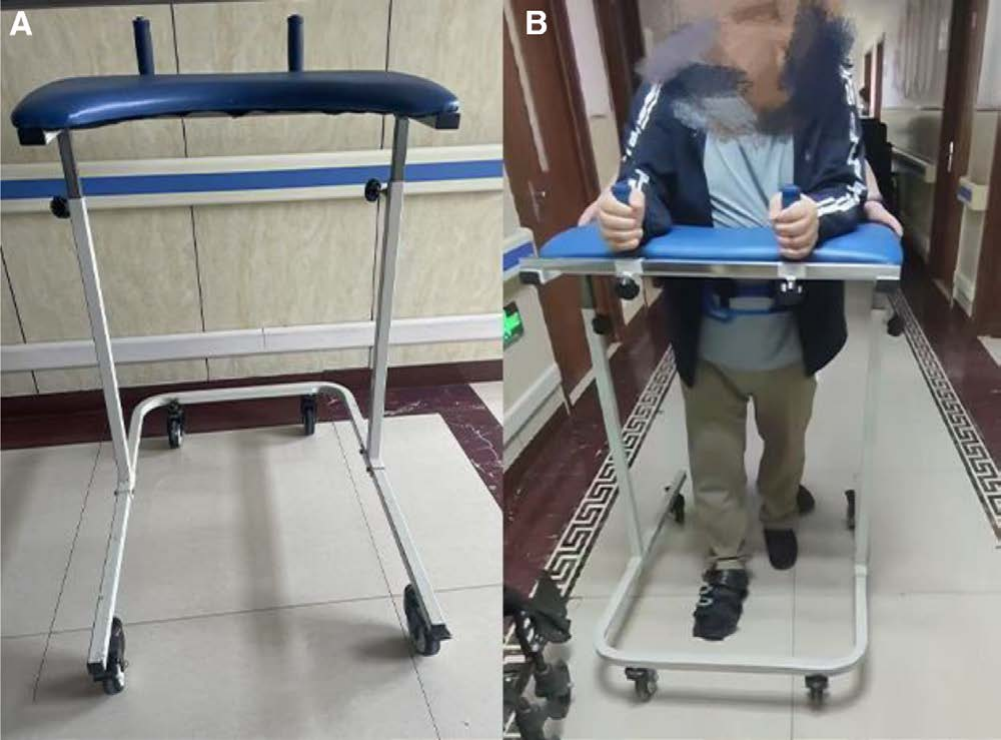
(A) Photograph of the device. (B) Photograph showing the patient walking with the assistance of the upright walker.

The treatment plan was adjusted after the DTI examination revealed severe damage to the corpus callosum fiber bundle. The high walk assistive device is used for forced walking training. High walk assistive device: Changzhou Kanghe Medical Co., Ltd. (model: KH-FZX-01). Dimensions: length 100 cm; width 82 cm; height 102 to 147 cm. Work surface pad height adjustment range: 45 cm. Handle spacing adjustment range: 0 to 55 cm. Frame structure. Work surface pad rated load capacity: 80 kg (Fig. 4). Patients use their upper-limb strength and the forward rolling of the wheels to propel themselves forward. The patient is wearing a harness around the waist. Throughout the training process, the therapist stands behind the patient to prevent them from falling. Training process details: The therapist presents a linear path and assists with controlling the walking speed. Initial walking speed: 0.28 to 0.5 m/s. Training walking speed: 0.8 to 1.3 m/s. The therapist guides the patient through swinging their limbs and torso from side to side and back and forth. The therapist synchronizes patient movements with the normal walking cycle as closely as possible. The patient can feel their body’s center of gravity shifting and how the affected side moves while walking. The training frequency was 20 minutes per session, twice a day, 38 days in total.

## 7. Follow-up and outcomes

During the initial assessment on March 3, 2025, the patient displayed total paralysis of their lower limbs. An assessment conducted on April 3, 2025, revealed improved upper limb function compared with pretreatment levels after 1 month of conventional rehabilitation therapy, but no significant changes in lower limb function. On April 8, 2025, the following was observed in surface electromyography (sEMG) signals from selected lower limb muscle groups: right gluteus maximus (affected side): root mean square (RMS) 15.414 μV. Left gluteus maximus (unaffected side): RMS 67.478 μV. Right rectus femoris: RMS 7.079 μV. Left rectus femoris: RMS 25.534 μV. Right tibialis anterior: RMS 9.375 μV. Left tibialis anterior: RMS 29.975 μV. Right gastrocnemius: RMS 7.312 μV. Left gastrocnemius: RMS 42.781 μV. The electrical signal value on the right side is clearly lower than that on the left. The sEMG data acquisition protocol is provided in the Supplementary Document, Supplemental Digital Content. Even after a week of continuous rehabilitation training, there was still no significant improvement in lower limb function in the patient. They were unable to walk unaided.

After conducting a DTI examination, the rehabilitation treatment plan was adjusted. On April 30, 2025, the patient will be able to walk 5 meters under supervision, but his gait will be unstable and abnormal (Supplemental Video). By May 14, 2025, the patient will be able to walk 20 meters independently, and their walking stability will have improved. However, foot drooping will still occur during gait. An sEMG test revealed marked increases in the RMS of the right gluteus maximus (the affected side), reaching 37.536 μV. It is 143.52% more than before. The RMS of the right rectus femoris is also marked increase, reaching 15.115 μV. It is 113.52% more than before. This suggests an improvement in the motor function of the right lower limbs of this patient. Meanwhile, the RMS values of the right tibialis anterior and right gastrocnemius muscles were 8.961 μV and 8.862 μV, respectively. The RMS values of the right tibialis anterior were −4.42% lower than before. The RMS values of the right gastrocnemius muscle increased by 21.20%. These values did not show a marked increase. The patient was discharged on May 23, 2025. He can now walk independently for 50 meters with clearly improved walking speed and stability. However, a right foot-drop gait persisted. Brunnstrom stages (right upper limb, hand, and lower limb): VI–V–IV. Standing balance: level 2/3. Holden walking function rating: level 4/5. Ten-meter walk test: 12.2 seconds. It was suggested that this patient’s balance and walking ability improved. Modified Barthel Index: 82 points. It was suggested that this patient’s activities of daily living improved. One month after discharge, a telephone follow-up revealed that, although precautions against falls were necessary, the patient was largely self-sufficient in daily activities and able to walk in the community.

Throughout the entire treatment process, the patient demonstrated excellent compliance and tolerated the training regimen well. There were no adverse events, such as falls, during the training sessions.

## 8. Discussion

After undergoing routine rehabilitation training, the patient’s lower limb motor function recovered slowly. An investigation into the cause of the slow recovery revealed that previous imaging studies of the patient did not show any damage to the corpus callosum. However, DTI revealed severe damage to the corpus callosum fiber tracts and the corticospinal tract. These 2 fiber bundles are highly correlated with motor function levels.^[9]^ The rehabilitation treatment plan was adjusted to include walking training with the assistance of a high walk assistive device. After receiving treatment, the patient was able to walk independently.

Following a cerebral infarction in the left frontal lobe near the midline, the surrounding brain tissue exhibited edema and an inflammatory response. This may further compress and damage the surrounding healthy neural tissue, thereby worsening neurological dysfunction. In clinical rehabilitation therapy, DTI is a key tool for revealing occult white matter lesions when patients show poor recovery outcomes and their clinical manifestations do not align with conventional imaging findings.

Walking is a rhythmic movement. It requires coordinated action of both sides of the body. This coordination is symmetrical and harmonious. Patients with lesions of the corpus callosum are more prone to gait abnormalities due to asymmetry in the use of information between the 2 sides of the body.^[10]^ Therefore, the treatment approach for patients should involve coordinating the use of both limbs and establishing a rhythmic environment. A previous study found that symmetrical and increased lateral sway-based walking training improved the walking function of patients with corpus callosum infarction.^[11]^ However, training with the high walk assistive device enables patients to walk with both lower limbs in an alternating, symmetrical manner. This strengthens the walking sequence in the brain, promoting the development of automatic movement patterns. Additionally, the high walk assistive device features 4 freely rolling wheels that place the patient in an unstable environment. This environment promotes appropriate alertness and enhances focus. This novel approach, which bypasses conventional training, may be more effective for patients with corpus callosum damage. It may facilitate better restoration of walking function. The training approach for gait rehabilitation in patients with poststroke hemiplegia has shifted from traditional methods to the integration of multimodal technology. There are many alternative gait rehabilitation training modes, such as robot-assisted gait training, virtual reality gait training, mirror therapy and movement observation training, hydrotherapy gait training, and functional electrical stimulation.

Motor deficits following a cerebral infarction are associated with the structural and functional integrity of the corticospinal tract.^[12]^ The corpus callosum integrates information from both cerebral hemispheres, playing a crucial role in coordinating movement.^[13]^ Reduced hemispheric sensorimotor connectivity exacerbates pseudoparalysis.^[14]^ Weakening of the use of information on the affected side can hide the true strength of the muscle. The affected limb is often mistaken for true paralysis, which hinders the patient’s recovery.^[15]^ This patient experienced severe damage to the corpus callosum fibers and corticospinal tracts. This resulted in a loss of motor command regulation from the left cerebral hemisphere to the lower right limb.

The brain possesses neuroplasticity.^[16]^ Strokes alter interhemispheric interactions indirectly. When motor function in 1 hemisphere is impaired, the other hemisphere can partially compensate by forming new neural connections.^[17]^ Research has found that corpus callosum synapses exhibit significant plasticity.^[18]^ A study found that hybrid-assisted limb gait training after a stroke promoted recovery of interhemispheric connectivity.^[19]^ The recovery outcome in this patient may be related to the brain’s white matter fiber remodeling. During the functional remodeling process, some latent motor control compensation methods may be activated.

In summary, using DTI to detect structural damage in the corpus callosum–corticospinal tract pathway can help ensure that rehabilitation interventions are precise. Because the patient did not undergo a second DTI scan, the mechanisms of recovery cannot be investigated. Additionally, there are possible roles of spontaneous recovery and concurrent conventional therapies. Therefore, our future research will involve conducting a multicenter randomized controlled clinical trial on forced walking training, while simultaneously using brain functional imaging techniques to conduct in-depth research on rehabilitation mechanisms.

## 9. Patient perspective

After his cerebral infarction, he was deeply concerned about his ability to walk independently and his prognosis, especially since his lower limbs were still paralyzed even after a month of rehabilitation. However, he was surprised to find that, after adjusting his rehabilitation therapy regimen for 2 weeks, he was able to walk slowly on his own. This gave him hope for recovery. He can now manage most daily activities independently, and he has reintegrated into social life. This has given him a sense of fulfillment and renewed confidence.

## Acknowledgments

We extend our gratitude to this patient and their family for their consent and cooperation.

## Author contributions

**Methodology:** Shiqiang Zhang.

**Software:** Shiqiang Zhang, Chencong Gu.

**Data curation:** Wei Feng, Xiao Wang.

**Writing – original draft:** Xiaodong Xu.

**Writing – review & editing:** Wenqian Bi.




