# Supplementary material for: Case report: Forced walking for treating lower limb paralysis after corpus callosum injury
Source: Medicine (Baltimore). 2026 May 22;105(21):e48767. doi: 10.1097/MD.0000000000048767 (PMC13200922; doi:10.1097/MD.0000000000048767)
Supplement: Supplementary file 1 [file medi-105-e48767-s001.docx]

Surface electromyography data acquisition protocol

Surface Electromyography Signal Acquisition: Surface EMG Acquisition and Transmission Device (Manufacturer: Beijing Changfeng Co., Ltd., Model: YW—Wireless). Bipolar differential electrodes are used with a center-to-center distance of two centimeters.

Relevant parameters: Sampling rate: 2,000 Hz. Filter processing: band-pass filtering: 20 Hz. Task protocol: Have the patient perform a maximum voluntary contraction. Calculate the root mean square (RMS) value. Calculate the RMS value for each cycle of muscle contraction tasks. Standardization Method: Take the average of three task cycles.

Gluteus Maximus Electrode Placement: Place the electrode at the midpoint along the line connecting the posterior superior iliac spine and the greater trochanter, and orient it parallel to the muscle fibers.

Rectus femoris electrode placement: The midpoint along the line connecting the anterior superior iliac spine and the upper border of the patella. It should be placed parallel to the thigh's longitudinal axis.

Tibialis anterior electrode placement: Located at the outer third of the line connecting the head of the fibula and the medial malleolus, at approximately the thickest part of the calf. It should be placed parallel to the longitudinal axis of the calf.

Gastrocnemius electrode placement: Located at the upper third of the line connecting the lower end of the popliteal fossa and the medial border of the Achilles tendon at the most prominent part of the muscle belly. It should be placed parallel to the longitudinal axis of the calf.
